# Treatment with CR500® improves algofunctional scores in patients with knee osteoarthritis: a post-market confirmatory interventional, single arm clinical investigation

**DOI:** 10.1186/s12891-023-06754-7

**Published:** 2023-08-12

**Authors:** Alessandra Colombini, Gianluca Doro, Enrico Ragni, Luca Forte, Laura de Girolamo, Fabio Zerbinati

**Affiliations:** 1 Laboratorio di Biotecnologie Applicate all’Ortopedia, IRCCS Istituto Ortopedico Galeazzi, Via R. Galeazzi 4, Milan, 20161 Italy; 2 Orthopedics and Traumatology Department, Humanitas Mater Domini, Varese, Italy; 3 Contrad Swiss SA, Lugano, Switzerland

**Keywords:** Knee osteoarthritis, Hyaluronic acid, Peptides, Synovial fluid, Algofunctional scores, Biochemical markers, Macrophages infiltration.

## Abstract

**Background:**

Knee osteoarthritis (OA) is a progressive and degenerative condition. Several pharmacological and non-pharmacological treatments are able to improve the OA symptoms and the structural characteristics of the affected joints. Among these, infiltrative therapy with hyaluronic acid (HA) is the most used and consolidated procedure for the pain management. The addition of skin conditioning peptides to HA promotes the cartilage remodeling processes and a better permeation of the HA-based gel containing a peptide mixture, CR500®. Furthermore, the topic route of administration is convenient over the routinely used intra-articular injective procedures. In this study, the effectiveness of CR500® was evaluated in terms of improvement of the algo-functional symptoms related to unilateral knee OA.

**Methods:**

38 mild and moderate OA patients were enrolled at a screening visit (V-1), treated at baseline visit (V1), and then continued the topical application of CR500® twice a week for 4 weeks, and followed-up for 3 visits (V2-V4) from week 2 to 4. Lequesne Knee Index (LKI) and Knee injury and Osteoarthritis Outcome Score (KOOS) were collected. Synovial fluid was collected and used for the quantification of neoepitope of type II collagen (C2C), C-terminal telopeptide of type II collagen (CTX-II), type II collagen propeptide (CPII), tumor necrosis factor alpha (TNFα) and HA. The expression of *CD11c* and *CD206* was evaluated on cell pellets.

**Results:**

Three patients were excluded, thus 35 patients were included in the analysis. The treatment with CR500® was safe and well tolerated, with 7.9% patients had mild adverse events, not related to the device. The LKI total score showed a significant decrease from V1 to V4. KOOS score also showed a significant improvement of patient condition at V2, V3 and V4 in comparison with V1 for all subscales, except for KOOS sport subscale which improved only from V3. At V1 a negative correlation among KOOS pain subscale values and C2C, CPII and TNFα levels was observed, as well as a positive correlation between KOOS pain subscale and *CD11c*/*CD206* ratio.

**Conclusion:**

CR500® is safe and appear to be effective in improving pain and function in OA patients during the 4 weeks of treatment.

**Trial registration:**

ClinicalTrials.gov ID: NCT05661162. This trial was registered on 22/12/2022.

**Supplementary Information:**

The online version contains supplementary material available at 10.1186/s12891-023-06754-7.

## Background

Osteoarthritis (OA) is the most common form of degenerative arthritis and one of the leading cause of disability in adulthood [1], affecting 303 million people [2], 4% of the world population [3], with a forecast of an increase in the affected population of 50% in the next 20 years [4]. The preferred locations are those most subjected to the load among which knee is the most affected joint.

Knee OA is a heterogeneous pathology, characterized by a complex and multifactorial nature, showing pain and stiffness in the affected joint. This multifactorial aetiology contributes to the broad variation in symptom presentation and treatment response that characterize patients [5, 6], and which constitutes a challenge for the identification of personalized and effective interventions [7].

OA is a progressive and degenerative condition, with unlikely regression and restoration of damaged structures; therefore, current modalities of disease management are aimed at symptom control, unless severity dictates the need for joint replacement surgery [8]. To date, there are no therapies that can prevent, reverse the progression from early to advanced OA, or that can induce a long-term improvement in symptoms [9]. Even total joint replacement surgery, necessary in the most advanced OA stages, cannot be considered as a curative treatment because of a residual functional limitation, which does not restore levels of mobility and functionality comparable to those of a healthy population [10]. However, there are several pharmacological and non-pharmacological treatments able to improve the OA symptoms and the structural characteristics of the affected joints [9]. These therapies, ranging from exercise and weight reduction [11], to intra-articular injections of corticosteroids, stem cells, or platelet-rich plasma [12] and other emerging compounds such as botulinum toxin [13] and ozone [14], showed various effectiveness. Among the therapeutic strategies for the OA treatment, infiltrative therapy is of fundamental importance and exploits various substances, among which the most used is hyaluronic acid (HA) [15]. In fact, intra-articular injection of HA is a well consolidated procedure for the knee OA management, and it is included in the OARSI guidelines as a non-surgical OA therapy, although with uncertain recommendation [16]. Nevertheless, it was reported that HA determined an improvement in pain symptoms for about 24–28 weeks, especially in movement, although in the long term follow-up it was not proven to be effective in modifying joint degeneration caused by OA [17, 18].

The rationale for the use of HA is based on the fact that it is a fundamental component of the synovial fluid (SF) to which it gives its viscoelastic properties [19]. From a biological point of view, it binds to cell receptors and modulate cell proliferation, apoptosis and migration [20–22]. HA is also a collagen stimulator, promoting tissue recovery [23] and is able reverse abnormal synthetic activity of OA subchondral bone [24]. HA is also able to modulate the inflammation and the recruitment of immune cells, as well as angiogenesis and tissue remodeling [25, 26].

Synovial fluid with normal HA concentration acts as a viscous lubricant during slow joint movements and as an elastic shock absorber during rapid joint movements. HA is also biodegradable, biocompatible, non-immunogenic and non-inflammatory, therefore it has been historically used in a large number of biomedical applications other than OA.

CR500® is a medical device based on sodium hyaluronate for topical use, therefore non-invasive, intended to attenuate the physiological degeneration of cartilage in OA. As suggested by the recent literature, skin conditioning peptides to promote a better permeation were added, resulting in a medical device containing sodium hyaluronate and two peptides, SH-Polypeptide-85 [27] and SH-Polypeptide-93 [28]. These human synthetic peptides were derived, respectively, from the Fibroblast Growth Factor 9 (FGF-9) and the Connective Tissue Growth Factor (CTGF). The presence of a peptide mixture and sodium hyaluronate facilitates the movement of joints and tendons for greater mobility and flexibility. Thanks to the peculiar properties of the peptides combination to modulate the cartilage remodelling processes [29, 30], CR500®, could exploit the synergy among HA, that provides a mechanical effect on joints, and peptides that together could improve some signaling and cellular response, swelling rate and porosity. These mechanical properties are directly associated to the gel performance and tissue response.

The aim of this single-arm, post-market confirmatory interventional clinical investigation was to evaluate the clinical effectiveness of CR500®, topically applied to the knee, in the attenuation of symptoms related to the physiological degeneration of cartilage, in patients affected by unilateral mild and moderate knee OA. To our knowledge, there are no studies reporting the efficacy of HA-based products for topical use in such context.

## Methods

### Patient enrolment and follow up

This was a confirmatory interventional, single arm, single-centre, post-marketing clinical follow-up study. The study was conducted according to the ISO 14155:2020, Good Clinical Practice guidelines, local laws and obligations and the World Medical Association Declaration of Helsinki.

The study was approved by the independent Ethics Committee of IRCCS Istituto Clinico Humanitas (HMD 913/20, date of approval 15/12/2020). Inclusion criteria were: age ≥ 18 years at the time of the enrolment, presence of unilateral knee OA of mild (score 1–4) or moderate (score 5–7) severity, assessed according to Lequesne Knee Index (LKI) [31] (Table 1).

**Table 1:** Osteoarthritis (OA) degree based on Lequesne Knee Index (LKI).

OA degree	LKI
None	0
Mild	1–4
Moderate	5–7
Severe	8–10
Very Severe	11–13
Extremely Severe	≥14

Exclusion criteria were: presence of bilateral OA and of other clinical conditions of the knee, immune system illnesses, uncontrolled systemic diseases, infective or inflammatory processes or damaged skin near the area of treatment, ongoing cutaneous allergies, serious and chronical pathological skin conditions and allergy to device components. Other exclusion criteria were: taking any other systemic or local therapy for the treatment of knee OA or other inflammatory diseases or painful states, drug and/or alcohol abuse, mental issues, pregnancy or breastfeeding.

Patients who were seen at the Orthopaedic Unit of the Mater Domini Clinic, Castellanza (Italy) between February 2021 and April 2022 were screened for the study. On a total of 650 patients, 38 satisfied the study criteria and were enrolled in the study.

Each patient underwent a screening visit (V-1), a baseline visit (V1), and 3 scheduled visits (V2-V4) on a weekly interval, from week 2 to week 4. Each subject, after having signed the informed consent form, entered the screening phase (V-1) that was performed within the 7 day period before the baseline visit (V1). The time of treatment for each enrolled patient was 4 weeks and the mean ± SD total time of inclusion in the study was 23.6 ± 1.6 days.

At each visit, the patients underwent the Knee injury and Osteoarthritis Outcome Score (KOOS) [32]; at V2 and V3 it was performed over the phone. At V1 the patients were asked to complete the LKI too. At V4 the patients were seen again by the physician to assess LKI, the safety and tolerability of CR500®, and concomitant therapies. At V1 and V4 the SF was collected through arthrocentesis.

All the patients received a diary record, from the baseline visit to V4, containing the instructions of use and two sections to be filled in in order to monitor health disturbances and potential concomitant intake of medications.

Satisfaction rate and tolerance to the treatment were both evaluated by a Likert scale, a type of psychometric response scale in which responders specify their level of agreement to a statement typically in five points: [1] Strongly disagree; [2] Disagree; [3] Neither agree nor disagree; [4] Agree; [5] Strongly agree [33].

### Treatment with CR500®

CR500® is a medical device, water and glycerin-based monodose gel, containing sodium hyaluronate and a peptide mixture in a 1.5 mL monodose vial. CR500® is formulated as follows: demineralized water, glycerin 99.8% PF, Propylene glycol, PEG-40 hydrogenated castor oil, preservative, carbomer, hyaluronic Acid HMW, xanthan gum, disodium EDTA, panthenol, sodium hydroxide, SH-Polypeptide-85 and SH-Polypeptide-93. The concentration of HA in CR500 monodose was 0.1%. It has been selected as effective to provide pain relief, as well as a reduction in the LKI in OA patients [34].

The treatment was performed at baseline visit (V1) and then after the first application, the treatment was repeated for two consecutive days per week, for 4 weeks, by the patients, at home. The patients were instructed to apply the product on the same two consecutive days every the week, (e.g. every Monday and Tuesday), such that each 2-day treatment period was separated by 5 days of no treatment. The patients had to apply the content of the single-dose vial to the painful area with a patch and keep it in place for 4 to 8 h. It was not necessary, nor recommended, to massage or spread the product gel.

### SF collection

Synovial fluid was collected at V1 and at V4 and stored at -80 °C until analyses. The frozen samples were thawed and centrifuged (10ʹ, 3900 g, 4 °C) to obtain a cell pellet to evaluate the gene expression of *CD11c* (pro-inflammatory macrophage marker) and *CD206* (anti-inflammatory macrophage marker). The cell-free supernatant of SFs were removed and used for the quantification of markers of type II collagen degradation (type II collagen (C2C) and C-terminal telopeptide of type II collagen (CTX-II)), type II collagen synthesis (type II collagen propeptide (CPII)), inflammation (tumor necrosis factor alpha (TNFα)) and HA.

### Gene expression analysis

The TRI Reagent® Solution (Applied Biosystems, Massachusetts, USA) was used for the extraction of RNA from cell pellets obtained from SF, following the protocol indicated by the manufacturer. The extracted RNA was eluted in 30 µl of water and reverse transcription was performed by using the iScript ™ gDNA Clear cDNA Synthesis Kit (Biorad, California, USA). Some samples were diluted 1: 3 because they were too viscous to be handled. The maximum allowed amount of template RNA (14 µl) was used for the reverse transcription of each sample.

Amplification of the cDNA by Real Time PCR was carried out using TaqMan chemistry (TaqMan® Gene Expression Master Mix and TaqMan ™ Gene Expression Assay, Applied Biosystems) and the StepOne Plus instrument (Applied Biosystems), following the thermal profile indicated by the manufacturer. To maximize the experimental output, the number of PCR cycles was extended to 60. The following commercial probes were used: Hs99999903_m1 (*ACTB*), Hs01015064_m1 (*CD11c*), Hs00267207_m1 (*CD206*). A volume of cDNA equal to 1:10 of the final reaction volume was added to each well. For each sample the readings were made in duplicate. Data were normalized using the *ACTB* expression and showed as ratio of the *CD11c* and *CD206* expression.

### Analysis of the protein markers in the SF

Synovial fluid samples depleted from cell pellets were used for the detection of biochemical markers. For the assay of C2C, CPII, CTX-II and TNFα the samples were pre-treated with 1 mg/mL of hyaluronidase (Sigma Aldrich, St Louis, MO, USA) in the presence of protease inhibitors. For the assay of C2C, CPII and TNFα the samples were used undiluted, while they were diluted 1:50 and 1:10.000 for the detection of CTX-II and HA, respectively.

The following commercial kits were used for the assay of the protein markers: Collagen Type II Cleavage Assay (C2C ELISA, IBEX, Quebec, Canada), Collagen Type II Synthesis Assay (CPII ELISA, IBEX), Serum Pre-Clinical CartiLaps® (CTX-II EIA, IDS, UK), Human TNF-alpha Quantikine HS (ELISA, R&D, Minnesota, USA) and Hyaluronic Acid Plus (TECOmedical AG, Switzerland). The range of detection for each analyte was reported from the manufacturer as follows: C2C 10–1000 ng/mL, CPII 0-2000 ng/ml, CTX-II 0-300 pg/ml, HA 0-1000 ng/mL and TNFα 0–10 pg/ml. The results of the protein quantifications were reported as the concentration in ng or pg per mL.

### Statistical analysis

Sample size calculation was performed supposing a minimum difference of 10% between LKI evaluated after treatment and at baseline, with a standard deviation (SD) equal to 1, a medium Pearson correlation of 30% between two timepoints and a type I error of 5% and 30 patients were considered sufficient to reach a statistical power greater than 80%. Moreover, planning to enrol a total of 38 subjects would have allowed for a 20% drop-out rate. Statistical power calculation was performed using SAS® software, v9.4.

Distribution of continuous variables were tested for normality by Shapiro Wilk tests. According to the result of this test, Kruskal Wallis test with Dunn’s post hoc test were used to assess differences in the clinical scores between study visits. In addition, correlation tests were performed using Spearman’s method to assess possible associations between clinical scores and protein content or gene expression data. P values < 0.05 were considered statistically significant. Data were analysed using Prism software v8.0 (Graphpad Prism, La Jolla, CA).

## Results

### Enrolled patients

**Fig. 1:**
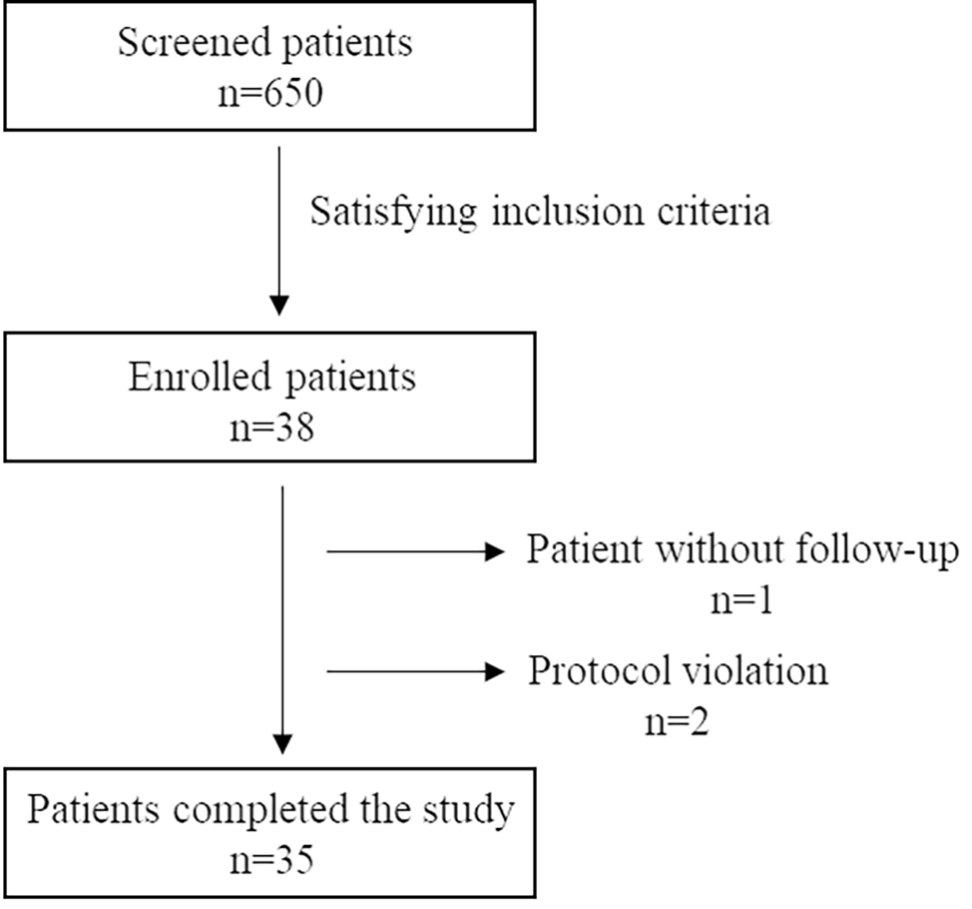
Flow chart of the enrolled patients

A total of 38 patients were enrolled in this study as planned by sample size calculation. All the patients were included in Safety Analysis Set (SAS) because all of them started the treatment. One patient (2.6%) was not included in the analysis because did not complete the Lequesne Knee Index (LKI) score. Two patients took medications to control pain at other sites than the target knee, representing a major protocol violation and were excluded from the analysis, for a total of 3 patients (7.9%). Figure 1 shows the flow chart of the 35 patients included in the analysis. Regarding visit status, V-1 and V1 were performed on 38 (100.0%) patients, whereas, V2, V3 and V4 were performed on 37 patients (97.4%).

### Demographic and other characteristic of the enrolled patients

Table 2 shows baseline characteristics of the enrolled patients.

**Table 2:** Baseline characteristics of the enrolled patients

		Overall (N = 38)
Age (year)		
	Mean ± SD	65 ± 12
	Median (IQR)	66 (57–74)
	Min-Max	26–83
Gender		
Male	N (%)	17 (45.9)
Female	N (%)	21 (54.1)
Ethnic group		
Caucasian	N (%)	37 (97.4)
Black	N (%)	1 (2.6)
Smoking status		
Non-smoker	N (%)	37 (97.4)
Smoker	N (%)	1 (2.6)
Abuse of drug and/or alcohol		
No	N (%)	38 (100.0)
Was pregnancy test performed?		
Yes	N (%)	2 (5.3)
NA/Male	N (%)	17 (44.7)
Menopausal status	N (%)	19(50)
Pregnancy test result		
NA	N (%)	37 (94.7)
Negative	N (%)	2 (5.3)

SD: Standard Deviation; IQR: Interquartile Range; NA: Not Applicable

Concerning the medical history, one (2.6%) patient had neoplasm inactive at the time of enrollment. Four patients (10.5%) had cardiovascular disorders, of which 3 had hypertension and one extrasystole. One patient (2.6%) suffered of hypothyroidism.

### Knee examination

Knee examination was performed at V-1, V1 and V4. At V-1 or V1 all the 38 patients were examined: 19 patients (50.0%) were symptomatic at their left knee and 19 (50.0%) at their right knee.

At V4, 37 patients had knee evaluation performed and had no allergic signs, lesions or other complications upon the use of CR500®.

### Safety and satisfaction rate assessment

For 33 patients (89.2%) the treatment was fully tolerated, for 3 (8.1%) was tolerated and just one (2.7%) had a neutral opinion.

Regarding the safety analysis, during the study only 3 patients (7.9%) had at least one adverse event (AEs), for a total of 7 AEs, and all unrelated to the device. No major issues were observed in term of vital signs (systolic and diastolic blood pressure, heart rate, and respiratory rate) and in physical examination, which could be linked to the study treatment.

Concerning the satisfaction rate after treatment with the product, 4 (10.4%) were very satisfied and 23 (62.2%) satisfied.

### Efficacy of the CR500®

**Fig. 2:**
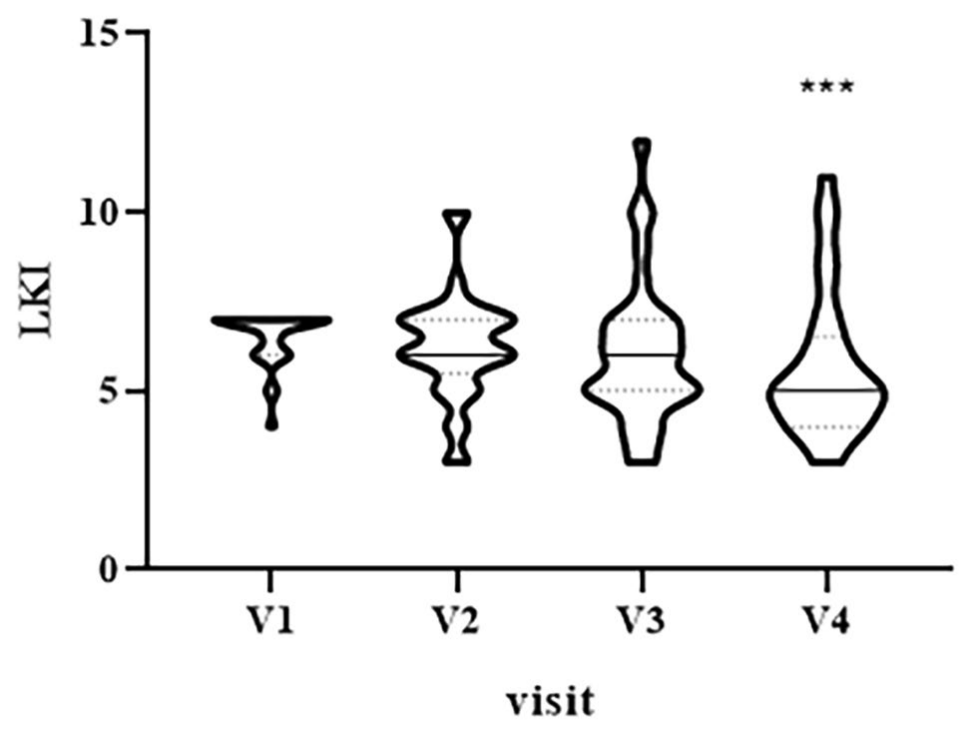
Violin plot showing the distribution of LKI values at different visits: V1 (baseline), V2 (two weeks), V3 (three weeks) and V4 (four weeks). ^***^p < 0.001, V1 vs. V4

The primary outcome, the LKI total score, showed a statistically significant decrease when measured from baseline to end-of study visit (median V1 = 7.0, interquartile range (IQR) [6–7], median V4 = 5, IQR [4-6.5], p = 0.0006, Fig. 2).

**Fig. 3:**
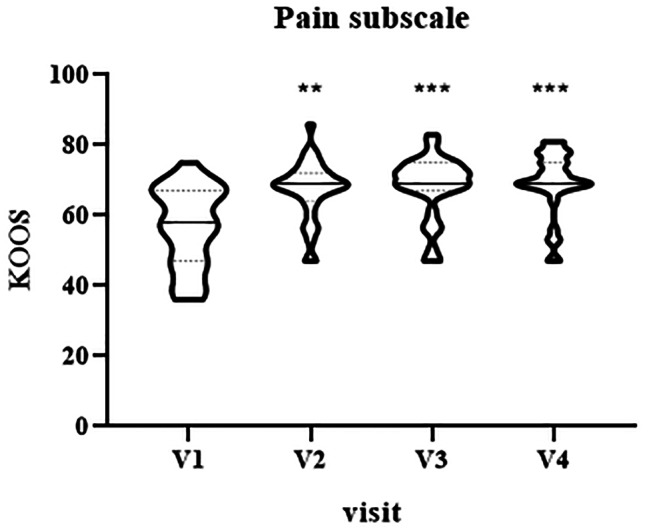
Violin plot showing the distribution of KOOS values, pain subscale, at different visits: V1 (baseline), V2 (two weeks), V3 (three weeks) and V4 (four weeks). ^**^p < 0.01, ^***^p < 0.001, V1 vs. V2-V4.

The analysis of KOOS pain subscale showed a statistical improvement of patients’ condition at two weeks (median V1 = 58, IQR [47–67] vs. median V2 = 69, IQR [64–72], p = 0.0026), three weeks (V1 vs. median V3 = 69, IQR [67–75], p = 0.0002) and four weeks (V1 vs. median V4 = 69, IQR [69–75], p = 0.0001) in comparison with baseline (Fig. 3).

**Fig. 4:**
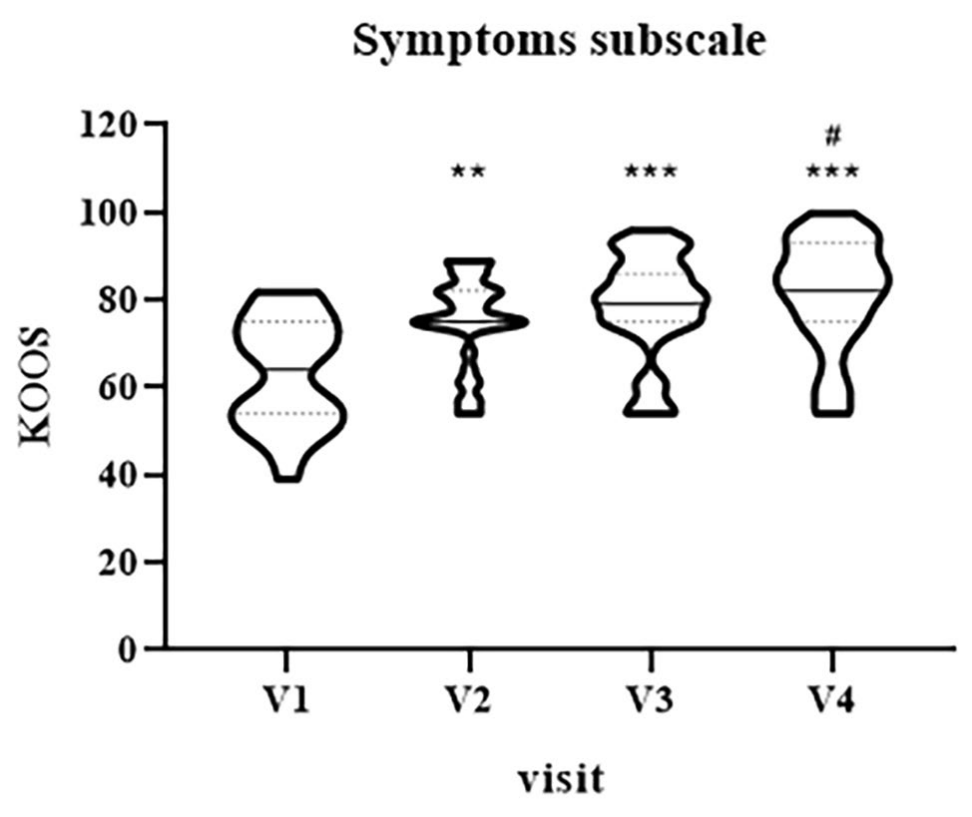
Violin plot showing the distribution of KOOS values, symptoms subscale, at different visits: V1 (baseline), V2 (two weeks), V3 (three weeks) and V4 (four weeks). ^**^p < 0.01, ^***^p < 0.001, V1 vs. V2-V4. ^#^p < 0.05, V2 vs. V4

As for pain, the evalution of KOOS symptoms subscale showed an improvement of scores from baseline to V4 at all the evaluated timepoints (Fig. 4, median V1 = 64, IQR [54–75] vs. median V2 = 75, IQR [75–82], p = 0.003; V1 vs. median V3 = 79, IQR [75–86] and vs. median V4 = 82, IQR [75–93], p < 0.0001; V2 vs. V4, p = 0.02).

**Fig. 5:**
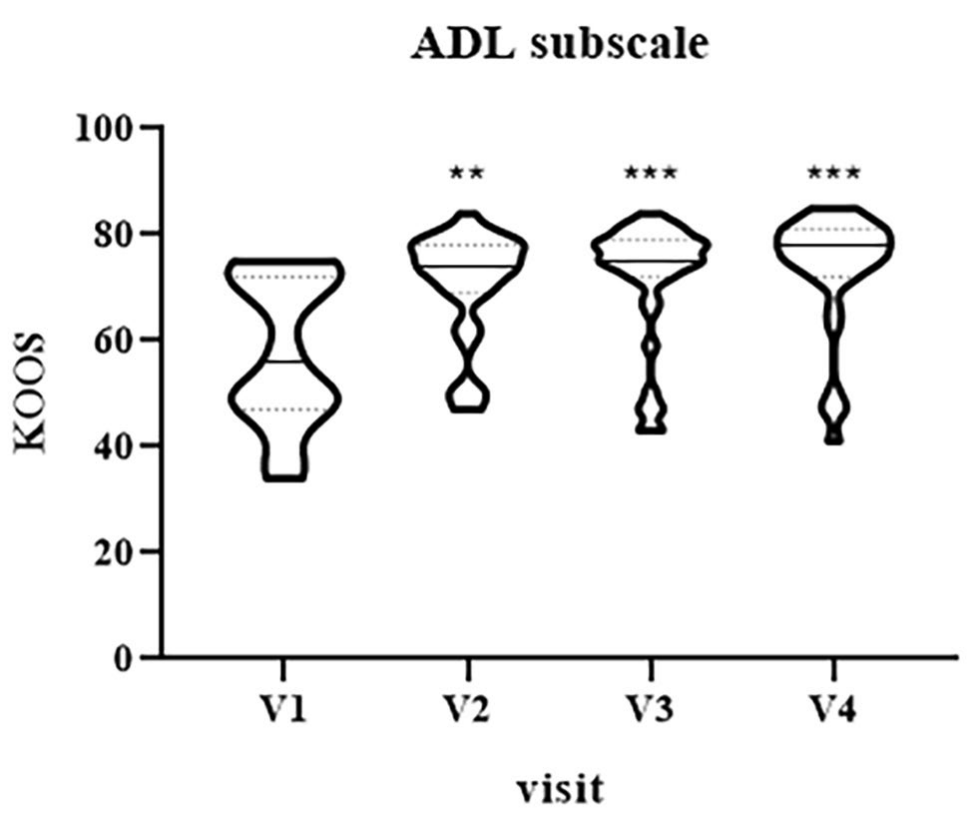
Violin plot showing the distribution of KOOS values, activities of daily living (ADL) subscale, at different visits: V1 (baseline), V2 (two weeks), V3 (three weeks) and V4 (four weeks). ^**^p < 0.01, ^***^p < 0.001, V1 vs. V2-V4.

KOOS activities of daily living (ADL) subscale values showed an improvement from baseline to V4 (Fig. 5, median V1 = 56, IQR [47–72] vs. median V2 = 74, IQR [69–78], p = 0.003; V1 vs. median V3 = 75, IQR [72–79], p = 0.00022; V1 vs. median V4 = 78, IQR [72–81], p < 0.0001).

**Fig. 6:**
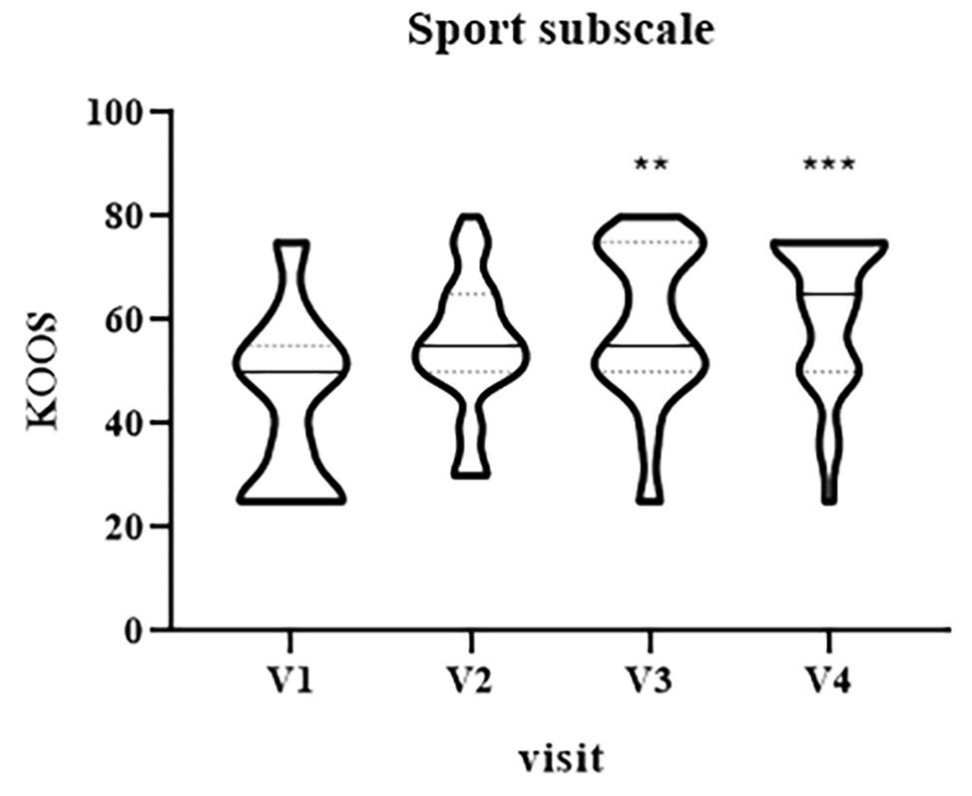
Violin plot showing the distribution of KOOS values, sport subscale, at different visits: V1 (baseline), V2 (two weeks), V3 (three weeks) and V4 (four weeks). ^**^p < 0.01, ^***^p < 0.001, V1 vs. V3 and V4, respectively

KOOS Sport subscale values showed an improvement only at V3 (median = 55, IQR [50–75], p = 0.001) and V4 (median = 65, IQR [50–75], p < 0.0001) in comparison with baseline (median V1 = 50, IQR [25–55]), as shown in Fig. 6.

**Fig. 7:**
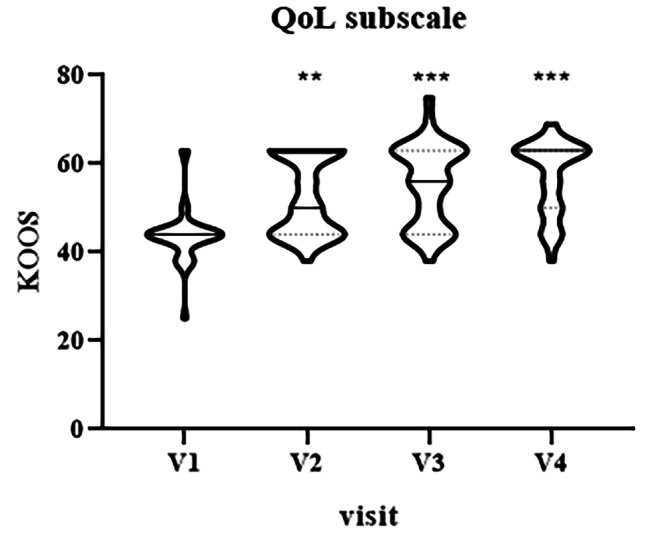
Violin plot showing the distribution of KOOS values, quality of life (QoL) subscale, at different visits: V1 (baseline), V2 (two weeks), V3 (three weeks) and V4 (four weeks). ^**^p < 0.01, ^***^p < 0.001, V1 vs. V2-V4.

Finally, quality of life (QoL) subscale showed an improvement at V2 (median = 50 IQR [44–63], p = 0.003), V3 (median = 56, IQR [44–63], p < 0.0001) and V4 (median = 63, IQR [50–63], p < 0.0001) in comparison with baseline V1 (median = 44, IQR [44–44]), Fig. 7.

### Analysis of biological markers in SF

SFs were collected at V1 from all the patients (n = 38). However, at V4, it was possible to collect samples from 2 patients only, due to the absence of extra SF from the rest of the cohort, for a total of 40 samples to analyze.

For what concerns the gene expresison analysis, putatively revealing the presence of inflammatory infiltrate, in 18 of 40 samples it was undetectable the expression of any of the three genes. In other 7 samples no expression of the housekeeping gene *ACTB* was observed probably due to the presence of a small amount of starting material, whereas one or both the other two genes of interest were detected, therefore preventing the appropriate evaluation of the expression of the target genes themselves. In 15 samples it was possible to determine the expression of the housekeeping *ACTB* gene and, at least, one of the two other target genes. Of those 15, for 11 samples it was possible to determine the *CD11c*/*CD206* ratio of expression (Supplementary Table 1), while in 4 it was not possible: the anti-inflammatory gene *CD11c* was not detected.

Regarding protein analysis, C2C, CTX-II and CPII were detected in all the samples, whereas TNFα was undetectable in 4 and HA in 3 samples at V1. The mean ± SD values for all the analysed markers are reported in Table 3.

**Table 3:** Levels of biochemical markers analyzed in synovial fluids at baseline (V1)

	C2C (ng/mL)	CTX-II (pg/mL)	CPII (ng/mL)	TNFα (pg/mL)	HA (mg/mL)
mean ± SD	153.7 ± 29.9	781.2 ± 411.7	585.8 ± 202.0	0.3 ± 0.2	3.3 ± 1.8

Type II collagen degradatiojn markers: neoepitope of type II collagen (C2C) and C-terminal telopeptide of type II collagen (CTX-II). Type II collagen synthesis marker: type II collagen propeptide (CPII). Inflammatory marker: tumor necrosis factor alpha (TNFα). Hyaluronic acid (HA).

The almost total lack of extra SF for samples at V4 made impossible to evaluate the modulation of the gene expression of the target genes or of the protein secretion mediated by the treatment. However, the biological markers were analysed in the light of the KOOS (pain subscale) to establish whether there was a correlation between the initial levels of markers and the clinical basal condition or the final clinical outcome. A negative correlation among KOOS pain subscale values at baseline and C2C, CPII and TNFα was observed, whereas a positive correlation between KOOS pain subscale and *CD11c*/*CD206* ratio emerged (Table 4).

**Table 4:** Correlation between the levels of biological markers analyzed in the synovial fluids and the KOOS (pain subscale) at baseline (V1)

	C2C	CTX-II	CPII	TNFα	HA	*CD11c*/*CD206* ratio
Spearman r	-0.47	-0.07	-0.41	-0.39	-0.11	0.77
95% CI	-0.70 to -0.15	-0.40 to 0.28	-0.66 to -0.079	-0.66 to -0.045	-0.45 to 0.25	0.33 to 0.93
p value	0.004^**^	0.691	0.014^*^	0.024^*^	0.529	0.004^**^

Type II collagen degradatiojn markers: neoepitope of type II collagen (C2C) and C-terminal telopeptide of type II collagen (CTX-II). Type II collagen synthesis marker: type II collagen propeptide (CPII). Inflammatory marker: tumor necrosis factor alpha (TNFα). Hyaluronic acid (HA). CI = confidence interval. ^*^p < 0.05, ^**^p < 0.01

A negative correlation was observed between KOOS pain subscale at V4 and C2C levels (Table 5).

**Table 5:** Correlation between the levels of biological markers analyzed in the synovial fluids and the KOOS (pain subscale) at 4 weekes (V4)

	C2C	CTX-II	CPII	TNFα	HA	*CD11c*/*CD206* ratio
Spearman r	-0.35	-0.26	-0.24	-0.13	0.24	0.14
95% CI	-0.62 to -0.015	-0.55 to 0.090	-0.53 to 0.11	-0.47 to 0.23	-0.13 to 0.54	-0.48 to 0.67
p value	0.036^*^	0.131	0.168	0.460	0.192	0.655

Type II collagen degradatiojn markers: neoepitope of type II collagen (C2C) and C-terminal telopeptide of type II collagen (CTX-II). Type II collagen synthesis marker: type II collagen propeptide (CPII). Inflammatory marker: tumor necrosis factor alpha (TNFα). Hyaluronic acid (HA). CI = confidence interval. ^*^p < 0.05

## Discussion

The main finding of the present study is that the topical application of CR500® is safe and suggest its effectiveness in the attenuation of symptoms in patients affected by mild to moderate knee OA.

No patients had a device-related AEs. HA with peptides embedded, are used in several biomedical applications with no major concerns. Patients were generally satisfied with the treatment, due to the non-invasive and convenient way of administration which could be a plus to guarantee satisfaction and compliance for the correct use.

The LKI total score, the primary outcome of the study, decreased significantly from V1 to V4. In addition, KOOS showed statistically significant improvements for all dimensions with respect to baseline visit. Except for sport subscale, all the other parameters from the KOOS questionnaire (pain, symptoms, ADL and QoL) significantly improved at V2, suggesting a quick efficacy of the treatment.

The biochemical evaluations on the synovial fluid showed a scarce amount of pro-inflammatory macrophages and that a lower cartilage remodelling correspond to a better patient’s pain score at baseline. Note that, at the last visit, extra synovial fluid was not present in the great majority of the patients, suggesting an action of the CR500® on its volume reduction.

Going deep in the possible mechanism of action of CR500®, the HA-peptide combination acts to modulate the degenerative/regenerative processes of knee joints. The HA is a well-known linear polysaccharide abundant in the connective tissue responsible for the viscoelastic quality of the synovial fluid which coats the surface of the articular cartilage. Thanks to its water-absorbing properties, it contributes to increase the lubricant and shock-absorbing capability of the SF. HA therapy provides anti-inflammatory effect through different pathways, e.g. by reducing the expression of pro-inflammatory mediators [35, 36], it prevents the degradation of cartilage and may promotes its regeneration by enhancing proteoglycans synthesis [36]. The peptides used in the formulation has been combined with HA to sustain specifically the anti-degenerative effects against arthritis damages. Peptides are short chains of amino acids with a wide range of biological actions [37], which are usually mediated through highly specific receptor-ligand interactions [38–40]. Indeed, a large number of regulatory cytokines are peptides [37], and peptides have been shown to exert a wide range of physiological effects [37, 38, 41, 42], including many with direct implications for OA treatment [43–46]. Two skin conditioning peptides, due to the synergy with the other components of the hydrogel formula, were selected to be added to CR500®. Peptides, indeed, are reported to enhance HA permeation on the skin [47], thereby promoting its absorption [48], as well as to be involved in cartilage remodelling processes. One of these peptides, SH-Polypeptide-85 [27], is a synthetic polypeptide derived from the human gene codifying for Fibroblast Growth Factor 9 (FGF-9), known to regulate the chondrocyte development [49, 50] and to attenuate the articular cartilage degradation [29]. SH-Polypeptide-93 [28] is a synthetic peptide derived from the human CTGF gene that orchestrates several signaling pathways [51], regulates the extracellular matrix composition [30] and the chondrogenesis [51, 52]. HA embedded with the aforementioned peptides may promotes tissues elasticity, strength, cartilage integrity and homeostasis, counteracting the OA symptoms by restoring intra-articular lubrication and viscoelastic properties of the SF, improving joint biomechanics and resulting in pain relief [53].

The advantage of using topical, hence non-invasive, and self-applied HA monodose gel allows to avoid intra-articular injection which is per se an invasive procedure as well as to eliminate the need to visit the clinic several times requiring the intervention of specialized staff.

Considering the improvement observed in the functional parameters evaluated in this study, it is plausible to hypothesize that the synergy of HA and peptides in the formulation ameliorate the health of the degenerated tissue. It was observed a decrease in the C2C levels quantified in the two patients from whom it was possible to collect the SF both at V1 and V4. C2C is a marker of type II collagen proteolytic degradation, which could be found in SF after knee damage independently on the type of injury [54]. Although not sufficient to extrapolate any statistical data, the reduction in the C2C protein marker strengthen the hypothesis that CR500® could contribute to reduce the physiopathological degeneration of the knee cartilage. At V4, the only negative correlation observed was between C2C levels and KOOS pain subscale: after 4 weeks, patients who felt better were those with low C2C basal levels, suggesting the importance of this parameter as biomarker of OA progression.

Unfortunately, it was not possible to evaluate the overall expression of genetic as well as biochemistry biomarkers after treatment in all the patients: 33 samples were screened at V1 for all the biochemical factors and 11 samples were completely analysed at V1 for gene expression. At V4 the volume of SF was averagely too low to be taken and analysed, and only the 2 aforementioned samples (500 µL-1 mL) were evaluated for protein biomarkers although not for genetic parameters. Although the lack of collection of SF at V4 from almost all the patients jeopardized the planned analysis, this suggest that the treatment itself could contribute to reduce the excess of SF production in knee OA processes, helping in the relief of pain generally associated with a consistent amount of SF which increase the intra-articular pressure as well as the local presence of catabolic and pro-inflammatory molecules [55]. Analysis of protein markers from patients at V1 gave little insights without the corresponding values from V4. As a consequence, no other major information were obtained on both biochemical markers of SF (besides C2C, CPII, CTxII, TNF-a and HA) and genetic parameters (*CD11c* and *CD206*) comparing V1 and V4. The analysis of biomarkers at V1 to establish whether there was a correlation between the initial levels of markers and the basal condition or the clinical outcome (pain subscale of the KOOS) revealed a negative correlation among KOOS pain subscale values and C2C, CPII and TNFα levels and, surprisingly, a positive correlation between KOOS pain subscale and *CD11c/CD206* ratio. These data suggest that patient with less pain can correlate with a lower cartilage remodelling (in terms of synthesis, CPII, as well as degradation, C2C) despite the presence of pro-inflammatory infiltrate (higher *CD11c*/*CD206* ratio). Nevertheless, a very scarce presence or absence of macrophages in some of the processed SFs was observed.

This study has some limitations. It is a single arm study: the absence of randomization cannot completely exclude the possibility of a natural and physiological improvement of general knee condition. However, data in accordance with some of the results that could be extrapolated from the analyses strongly suggest that it occurs thanks to the treatment itself. Although statistically adequate, the quite low number of subjects enrolled for this study suggests to perform further studies to confirm the efficacy of the product in a larger cohort of patients, including also a control cohort of untreated subjects. Other limitations are represented by the lack of OA imaging classification and by the lack of a medium- (3 or 6 months) as well as long-term follow-up: it would be important to evaluate whether the positive effects of CR500® are maintained even after the interruption of its use.

## Conclusions

This study allowed to conclude that CR500® single gel is safe and well tolerated by all patients exposed to the treatment. Internationally validated subjective scales indicated that treatment with the investigational product correlated with a marked and statistically significant improvement in knee pain and function. These results suggest that CR500® has the potential to attenuate the symptomatology related to the osteoarthritic processes starting from the 2nd week of treatment.

### Electronic supplementary material

Below is the link to the electronic supplementary material.

Supplementary Material 1

## Data Availability

The data that support the findings of this study are openly available at: https://osf.io/skb9r/?view_only=6139266ad08647e2abaa4550004df3fe.

## List of Abbreviations

OA: Osteoarthritis
HA: Hyaluronic acid
SF: Synovial fluid
LKI: Lequesne Knee Index
KOOS: Knee injury and Osteoarthritis Outcome Score
C2C: Neoepitope of type II collagen
CTX-II: C-terminal telopeptide of type II collagen
CPII: Type II collagen propeptide
TNFα: Tumor necrosis factor alpha
IQR: Interquartile range
