# Supplementary material for: Treatment with CR500® improves algofunctional scores in patients with knee osteoarthritis: a post-market confirmatory interventional, single arm clinical investigation
Source: BMC Musculoskelet Disord. 2023 Aug 12;24:647. doi: 10.1186/s12891-023-06754-7 (PMC10422714; doi:10.1186/s12891-023-06754-7)
Supplement: Supplementary file 1 — Supplementary Material 1 [file 12891_2023_6754_MOESM1_ESM.docx]

Supplementary Table 1. *CD11c*/*CD206* ratio of expression.

| **Sample Name** | **CD11c/CD206 ratio** |
| --- | --- |
| Sample 6 | 61.2 |
| Sample 11 | 7.1 |
| Sample 16 | 4.8 |
| Sample 17 | 6.3 |
| Sample 18 | 19.9 |
| Sample 29 | 71.5 |
| Sample 30 | 21.9 |
| Sample 33 | 112.0 |
| Sample 34 | 22.2 |
| Sample 35 | 79.8 |
| Sample 36 | 47.5 |
